# Subtle Alterations in Spatial Memory Induced by Amyloid Peptides Infusion in Rats

**DOI:** 10.3389/fnagi.2018.00018

**Published:** 2018-01-30

**Authors:** Priscila Tavares Macêdo, Antônio C. Q. Aquino, Ywlliane S. R. Meurer, Luiz E. M. Brandão, Clarissa L. C. Campêlo, Ramon H. Lima, Marcos R. Costa, Alessandra M. Ribeiro, Regina H. Silva

**Affiliations:** ^1^Memory Studies Laboratory, Physiology Department, Universidade Federal do Rio Grande do Norte, Natal, Brazil; ^2^Brain Institute, Universidade Federal do Rio Grande do Norte, Natal, Brazil; ^3^Behavioral Neuroscience Laboratory, Pharmacology Department, Universidade Federal de São Paulo, São Paulo, Brazil; ^4^Laboratory of Neuroscience and Bioprospecting of Natural Products, Department of Biosciences, Universidade Federal de São Paulo, São Paulo, Brazil

**Keywords:** neurodegenerative disease, β-amyloid peptide, barnes maze, navigation, strategy

## Abstract

The cause of Alzheimer’s disease (AD) remains uncertain. The accumulation of amyloid peptides (Aβ) is the main pathophysiological hallmark of the disease. Spatial deficit is an important initial sign of AD, while other types of memory impairments that appear in later stages. The Barnes maze allows the detection of subtle alterations in spatial search by the analysis of use of different strategies. Previous findings showed a general performance deficit in this task following long-term (35 days) infusion of Aβ, which corresponds to the moderate or severe impairments of the disease. In the present study, we evaluated the effects of a low-dose 15-day long treatment with Aβ peptides on spatial and non-spatial strategies of rats tested in the Barnes maze. Aβ peptides (0.5 μL/site/day; 30 pmoL solution of Aβ1–40:Aβ1–42 10:1) or saline were bilaterally infused into the CA1 (on the first treatment day) and intraventricularly (on the following 15 days) in 6-month-old Wistar male rats. Aβ infusion induced a deficit in the performance (increased latency and distance traveled to reach the target compared to saline group). In addition, a significant association between treatment and search strategy in the retrieval trial was found: Aβ group preferred the non-spatial search strategy, while saline group preferred the spatial search. In conclusion, the protocol of Aβ infusion used here induced a subtle cognitive deficit that was specific to spatial aspects. Indeed, animals under Aβ treatment still showed retrieval, but using non-spatial strategies. We suggest that this approach is potentially useful to the study of the initial memory deficits in early AD.

## Introduction

Alzheimer disease (AD) is a neurodegenerative disorder characterized by atypical neural activity, dysfunction and loss of synapses and neurons, accompanied by progressive cognitive decline (Braak et al., 1992; West et al., 1994; Eustache et al., 2004; Sadek et al., 2004). The main pathophysiological hallmarks of AD are amyloid plaques and neurofibrillary tangles in the brain tissue (Serrano-Pozo et al., 2011). The exact cause of AD remains unknown, although the accumulation of amyloid peptides has been suggested as the cause of cytotoxicity. This accumulation is the basis of the amyloid cascade hypothesis for AD and precedes the first manifestations of cognitive decline (Braak and Braak, 1991; Hardy and Selkoe, 2002; Desikan et al., 2013; Sperling et al., 2011, 2014).

Recent studies have investigated human spatial learning and memory in virtual reality environments (Iaria et al., 2009; Head and Isom, 2010; Plancher et al., 2010). This approach has been useful to show age-related cognitive hippocampal dysfunctions, similarly to studies in rodent models (McNaughton et al., 1989; Poucet et al., 1991; McDonald and White, 1993; Pouzet et al., 2002). The results highlight the importance of protocols for early detection of such impairment (Foster et al., 2012). Further, spatial deficits detected in navigation tasks have been proposed as an important initial sign of AD, and this feature distinguishes this condition from other types of age-related cognitive impairments (Lithfous et al., 2013). In addition, deficits in other types of memory usually arise in later stages of the disease progression (Sá et al., 2012; Tarawneh and Holtzman, 2012).

Spatial tasks have been employed to investigate cognitive decline in rodents, and some of the tasks allow the detection of subtle changes in spatial behavior. This feature is relevant to the evaluation of mild hippocampal impairment, such as the initial degenerative process in animal models of AD. The Barnes maze is as spatial task in which the animals are trained to reach a hidden shelter guided by visual cues (Barnes, 1979; Barnes et al., 1980, 1989, 1990; Harrison et al., 2006; O’Leary and Brown, 2012). In this task, memory is evaluated by latency and distance to reach the target, time in target quadrant and number of errors (Sunyer et al., 2007; Attar et al., 2013; Rojanathammanee et al., 2013). In addition, subtle alterations in spatial search can be detected by the analysis of different search strategies to solve the task (Harrison et al., 2006). This behavioral task has been used to investigate spatial memory impairment in models of neurodegenerative conditions, including AD (Pompl et al., 1999; Reiserer et al., 2007; O’Leary and Brown, 2009).

Brain infusion of Aβ peptides is a well recognized rodent model of AD (Kowall et al., 1991; Nitta et al., 1994; Frautschy et al., 1996; Yamada and Nabeshima, 2000; Kang et al., 2015). However, most of the Aβ infusion studies focus the intermediate or late stages of the disease (Dickey et al., 2003, 2004; Palop et al., 2003; Blanchard et al., 2008, 2009), an approach that would diminish the contribution to the efficiency of potential neuroprotective treatments. In general, the protocols are conducted with large concentrations or long treatment durations (Han et al., 2011; Tran et al., 2011; Cioanca et al., 2013). To our knowledge, only one study investigated the effects of Aβ treatment in mice tested in the Barnes maze. This work showed a general performance deficit following long-term (35 days) infusion, without evaluation of different strategies (Morzelle et al., 2016). Here, we used a protocol of Aβ infusions that induces subtle memory deficits, which would be similar to initial stages in humans. The effects of this treatment were investigated in Barnes maze general performance and use of spatial or non-spatial search strategies. We hypothesize that the protocol of Aβ infusion used here will induce subtle deficits in the maze performance, specifically in the use of spatial strategies.

## Materials and Methods

### Animals

Seventeen six-month-old male Wistar rats (350–500 g) were kept in four or five animals per cage (30 × 37 × 16 cm) under controlled conditions of temperature (25 ± 1°C) and luminosity (12/12 h light/dark cycle, with lights turned on 7:00 a.m. and turned off 7:00 p.m.). Food and water were provided *ad libitum*. All rats were handled during 3 days, at least 5 min per day, previously to the beginning of the experiments. All procedures were in accordance to the Brazilian Law for the use of animals in scientific research (law 11.794) and approved by the local ethics committee (CEUA/UFRN, protocol no. 50/2014). All efforts were made to minimize pain, suffering or animal discomfort. Before the procedures, animals were allocated in the experimental room for habituation. The behavioral experiments were conducted in the mornings, beginning at 8 a.m.

### Stereotaxic Surgery

After anesthesia with ketamine (100 mg/kg) and xylazin (50 mg/kg), the animals were submitted to stereotaxic surgery and cannulas were implanted bilaterally into dorsal hippocampus subregion CA1 (AP: −4.2 mm; LL: ±3.0 mm and DV: −2.5 mm) and one in the lateral ventricle (i.c.v.; AP: −1.0 mm; LL: ±1.8 mm and DV: −3.3 mm). Right or left hemispheric location was randomized among rats. The coordinates were chosen according to a rat brain atlas (Paxinos and Watson, 2007) and adjusted considering age. The microinjections were performed with an infusion pump and a 10 μL microsyringe (Hamilton Company, Reno, NV, USA) connected to a 26-gauge steel needle into the brain sites.

### Beta-Amyloid (Aβ) Infusion

Animals received infusions (0.5 μL at the rate of 1 μL/min) of Aβ or vehicle i.c.v. and CA1 (bilateral) on the first day, followed by 14 daily i.c.v. infusions. Animals in the Aβ group (*n* = 9) received a 1:10 solution of 1–42 and 1–40 Aβ peptides (Iwatsubo et al., 1994; Suzuki et al., 1994; Gravina et al., 1995), equivalent to 30 pmoL per day, diluted in 0.9% saline. Before the beginning of infusions, each Aβ peptide solution was separetaly heated at 37°C for 3 days (Pike et al., 1991). Afterwards, 1–42 and 1–40 Aβ peptides (Sigma Chemical Co. St. Louis, MO, USA) were put together and left at 37°C for 24 h. Control animals receive the same volume of saline (*n* = 5). Two saline and one Aβ animals were excluded from the analyses because they did not reach the target in the probe session.

### Barnes Maze

The Barnes maze is a spatial learning apparatus in which the animals search for an escape box (Barnes, 1979). The maze is a dark circular platform (120 cm diameter), elevated 90 cm from the floor, containing 20 holes (10 cm in diameter) disposed circularly at the edge of the platform. One of the holes (target hole) is connected to an escape box (10 × 10 × 15 cm). There were visual cues on the walls, located 50 cm distant from the apparatus. During the behavioral sessions, the lights were turned on (420 lux) to increase escape motivation. All experimental sessions were recorded by a video camera placed above the apparatus and analyzed with a video-tracking software (ANY-maze, Stoelting, USA).

**Figure 1:**
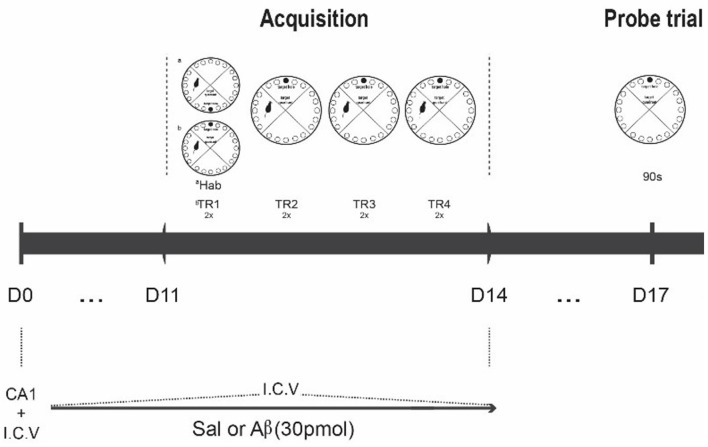
Experimental design. Rats were infused with saline or Aβ solution for 15 days (D0 to D14); at D0, CA1 and I.C.V. infusion; from D1 to D11, only I.C.V. infusion. After the 11th infusion, the rats were submitted to the habituation phase (Hab), followed by acquisition phase (two series of four two-trial daily trainings, Tr1 to Tr4, D11 to D14). On the 3rd day after the last training day, they were tested for task retrieval (90 s probe trial, D17). The perfusion for immunohistochemistry was conducted 1 h after the probe trial.

In the habituation session, the animals were allowed to freely explore the apparatus during 150 s or until the entrance in escape box. Afterwards, the animals were submitted to a set of four daily training sessions, each one with two trials. The inter-trials interval was the duration of cleaning the apparatus (with a 5% alcohol solution). The first training session was performed immediately after the habituation. All trials lasted 300 s or until the animals reached the escape box. However, if the rats did not reach the target hole, the experimenter gently guided the animal towards it at the end of the trial. After reaching the escape box, animals remained inside for 60 s. The escape box is always located in the same place during the training set, but not during the previous habituation session. Retrieval of spatial learning was evaluated in the probe session, which was conducted 3 days after the last training day. The procedure was similar to the training trials, but the escape box was removed. At the beginning of each session, the animals were placed in an opaque container at the center of the maze. The container was then pulled up, and the animal was released to explore the maze. Between animals, the maze was cleaned and rotated to avoid odor cues (Figure 1).

The parameters analyzed were the percentage of time in the quadrants of the maze, the latency to reach the target hole, the distance traveled until reaching the target hole and the number of errors. We also analyzed search strategies (the kind of search performed to reach target), and analysis was conducted on the basis of previous studies (Bach et al., 1995; Harrison et al., 2006), with minor modifications. The search strategies were categorized into three types: spatial (or direct), serial or random (the two latter were considered non-spatial strategies). One search was computed each time the animal visited the target hole in the probe trial. The direct (spatial) strategy was computed when the animal moved directly to the target hole or to an adjacent hole before visiting the target hole. The serial strategy was computed when there were visits to at least three sequential holes in clockwise or counter-clockwise direction previously to the visit to the target hole. The random strategy was computed when at least three visits before the target hole happened in an unsystematic manner, i.e., the animal visited non-adjacent holes before visiting the target. The type of strategy was analyzed by the following approaches: (1) initial and last search (the strategy used to reach the target for the first and the last time in the probe trial); (2) total preference in the probe trial (the percent of use of each strategy across the whole probe trial (the number of times the animal used a certain strategy/total number of searches); and (3) the mean percentage of preferred strategy in each group.

### Data Analyses

We used Shapiro-Wilk test to check data distribution. We applied one-way ANOVA, one- or two-sample *t*-tests according to the parameter analyzed. For analyses across sessions, we performed repeated-measures ANOVA. Bonferroni *post hoc* test was applied when necessary. Fisher’s exact test (*x*^2^) was used for the analysis of the association between treatment (Aβ/saline) and preferred strategy, as well as the magnitude of this association in 95% confidence interval (*odds ratio*, OR, 95% CI). Data are shown as mean ± SEM. We determined the level of significance at *p* < 0.05.

## Results

**Figure 2:**
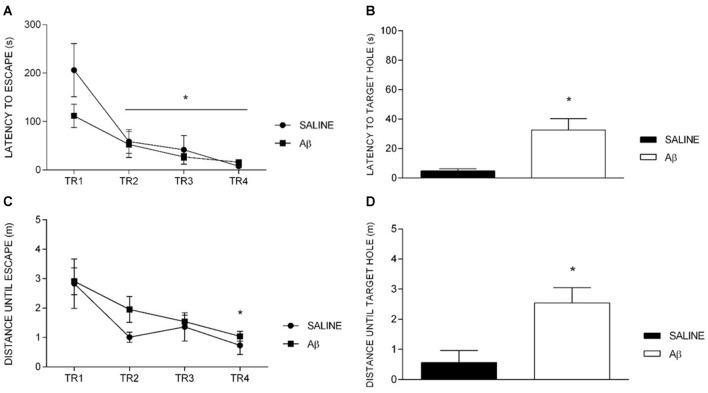
Latency and distance until escape in training (TR) sessions and until target hole in probe trial. **(A,C)** Effect of session for both groups (repeated measures two-way ANOVA, Bonferroni *post hoc*); *p < 0.05 compared to TR1. **(B,D)** Unpaired *t*-test; *p < 0.05 compared to SALINE. Mean ± SEM.

The latency to reach the escape hole diminished over training sessions in both groups, indicating similar learning curves (*F*_(1.7,21.2)_ = 16.688, *p* < 0.001, Bonferroni *post hoc*; Figure 2A). Indeed, we did not find differences between groups (*F*_(1,12)_ = 1.592, *p* = 0.231) or factors interaction (*F*_(1.7,21.2)_ = 2.103, *p* = 0.151). However, in the probe trial, Aβ group displayed longer latency to reach the target hole for the first time (*t*_(12)_ = −2.679, *p* = 0.02; Figure 2B).

We observed the same pattern of results for the distance traveled until the escape hole across the training sessions (session effect [*F*_(3,36)_ = 9.638, *p* < 0.001], without group effect [*F*_(1,12)_ = 1.005, *p* = 0.336] or interaction [*F*_(3,36)_ = 0.509, *p* = 0.678], Bonferroni *post hoc*; Figure 2C). On the probe trial, we found that Aβ groups showed longer distance traveled until the target than saline (*t*_(12)_ = −2.636, *p* = 0.022; Figure 2D).

**Figure 3:**
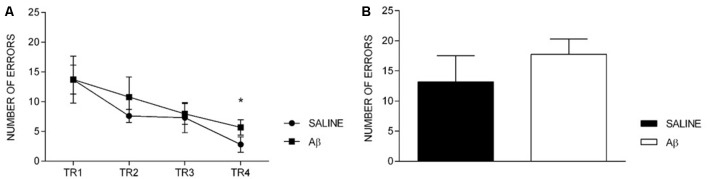
Number of errors in training sessions (TR, **A**) and probe trial **(B)**. **(A)** Effect of session for both groups (repeated measures two-way ANOVA, Bonferroni *post hoc*); *p < 0.05 compared to TR1. **(B)** Unpaired *t*-test; *p* > 0.05. Mean ± SEM.

The number of errors reduced across the training sessions for both groups (*F*_(3,36)_ = 5.419, *p* = 0.004, Bonferroni *post hoc*; Figure 3A). In addition, we did not find differences between groups (*F*_(1,12)_ = 0.573, *p* = 0.464) or factors interaction (*F*_(3,36)_ = 0.218, *p* = 0.883). Likewise, on the probe trial, there was no difference between the groups (*t*_(12)_ = −0.985, *p* = 0.344; Figure 3B).

**Figure 4:**
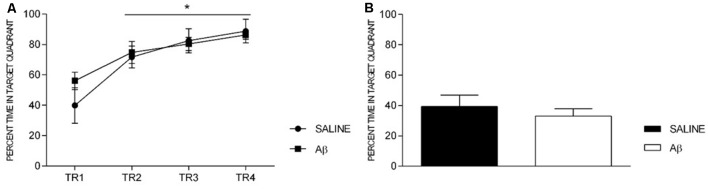
Percent time in target quadrant in training sessions (TR, **A**) and probe trial **(B)**. **(A)** Effect of session for both groups (repeated measures two-way ANOVA, Bonferroni *post hoc*); *p < 0.05 compared to TR1. **(B)** Unpaired *t*-test; *p* > 0.05. Mean ± SEM.

Additionally, both groups showed increased time in the target quadrant across trainings (*F*_(3,36)_ = 14.954, *p* < 0.001, Bonferroni *post hoc*; Figure 4A), without treatment effect (*F*_(1,12)_ = 0.416, *p* = 0.531) or factors interaction (*F*_(3,36)_ = 0.946, *p* = 0.429). On the probe trial, there was no difference between groups (*t*_(12)_ = 0.736, *p* = 0.476; Figure 4B).

**Figure 5:**
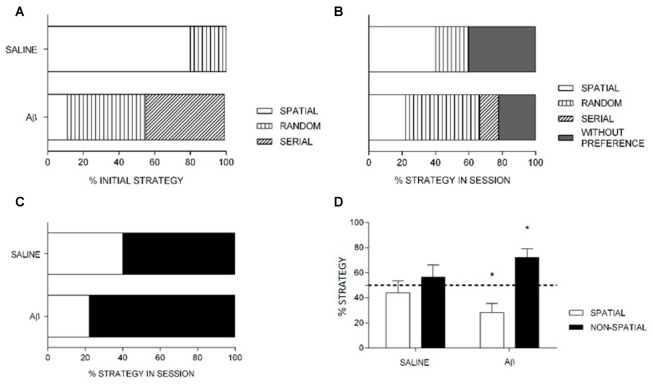
Percentage of strategy use at initial choice **(A)** and in the total **(B,C)** probe trial. **(A,B)** Saline had increased initial **(A)** and total **(B)** spatial strategy choice compared to Aβ group. **(C)** The preferential use of spatial strategy by saline is sustained when spatial, non-spatial and without preference are grouped into non-spatial strategy. **(A–C)** Data are percentage of strategy compared between the groups. Fisher’s exact test, *x*^2^, *p* < 0.05. **(D)** Percentage of spatial and non-spatial strategies (mean + SEM) differed from chance for Aβ, but not for saline group (one-sample *t*-test). *p < 0.05 compared to chance (dashed line).

We found a significant effect of treatment on the search strategy in the probe trial. Saline group preferred the spatial strategy as initial choice compared to Aβ group (Fisher’s exact test, $x_{\left(1\right)}^{2}$ = 95.998, *p* < 0.001; Figure 5A). The probability that saline group had used the spatial strategy was 32 times higher than Aβ group (OR 32.36, 95% CI: 14.61–71.69).

Considering the search strategies in the whole probe session, the Aβ group used less spatial search strategy compared to saline (Fisher’s exact test, $x_{\left(1\right)}^{2}$ = 20.276, *p* < 0.001; OR 5.091, 95% IC 2.456–10.553, Figure 5B). In addition, the analysis of groups by strategy in the whole probe session (including animals that preferred random or serial strategies and had no preference into the non-spatial category), Aβ group sustains the preferential use of non-spatial search strategies compared to saline animals (Fisher’s exact test, $x_{\left(1\right)}^{2}$ = 7.574, *p* = 0.009; OR 2.364, 95% IC 1.272–4.392; Figure 5C).

We also analyzed the last choice, i.e., the strategy used to reach the target for the last time in the probe session. We found that the Aβ group persevered on non-spatial strategies, while saline-treated animals changed preference towards the end of the probe session. Indeed, within subjects analyses (first vs. last choices) confirmed the change of preference in saline group ($x_{\left(1\right)}^{2}$ = 72.000, *p* < 0.001; OR, 16.000, 95% CI: 8.002–31.994), and the persistence of preference for non-spatial strategies in the Aβ group ($x_{\left(1\right)}^{2}$ = 4.391, *p* = 0.056; OR, 0.438, 95% CI: 0.200–0.961). In addition, the use of strategies as last choice did not differ between groups ($x_{\left(1\right)}^{2}$ = 3.196, *p* = 0.082; OR 2.045, % CI: 0.924–4.529). In Table 1, we show the percent of animals that show spatial and non-spatial preferences for the first three and the last choice in the probe session. We show four choices because it was the minimum number of strategies used by a subject in the probe session.

**Table 1:** Percentage of strategies used at the first three and the last choices in the probe trial.

Group	Type of strategy	Choice in the probe session			
		1st	2nd	3rd	Last
Saline	Spatial	80.00	60.00	40.00	20.00^#^
	Non-spatial	20.00	40.00	60.00	80.00^#^
Aβ	Spatial	11.11*	44.44	22.22	22.22
	Non-spatial	88.89*	55.56	77.78	77.78

Fisher’s exact test, *x*^2^, *p* < 0.05: *compared to saline; ^#^compared to first choice.

The comparison of means of percentage of strategies in the groups in the whole probe session showed that saline group used possible search strategies similarly (spatial, *t*_(4)_ = −0.636, *p* = 0.559; non-spatial, *t*_(4)_ = 0.685, *p* = 0.531), while the Aβ group preferred non-spatial search strategies (spatial, *t*_(8)_ = −3.020, *p* = 0.017; non-spatial, *t*_(8)_ = 3.121, *p* = 0.014; Figure 5D).

## Discussion

We observed that both groups showed reduced distance and latency to reach target and number of errors, and increased percent time in the target quadrant across the trainings, indicating that all the animals learned the task (Barnes, 1979; Pompl et al., 1999). Moreover, we found that animals that received Aβ peptides showed impairments when the latency and distance to reach the target were analyzed in the probe trial. However, no differences were found to the other performance parameters (number of errors and percent time in the target quadrant) in the probe session, suggesting that Aβ animals showed some extent of retrieval. In addition, we found that Aβ-infused animals preferentially used non-spatial search strategies, corroborating an impairment in spatial retrieval, despite the performance was not completely impaired. Finally, there were no differences in the distance traveled in the maze between saline and Aβ groups, indicating that Aβ peptides infusion did not modify motor activity (data not shown).

Our data are in line with previous studies that suggest that the amyloid aggregation impairs hippocampal connectivity, which is necessary for spatial navigation and for strategy choice (Pouzet et al., 2002; Savonenko et al., 2005; O’Leary and Brown, 2009). In the present study, when the search strategies to reach target were analyzed, saline group preferred the spatial strategy to the non-spatial strategy, while Aβ animals had the opposite preference. This outcome suggests that the deficit promoted by beta-amyloid was revealed by the kind of search navigation, which has modified the spatial performance. Of note, when the analysis of search strategies was performed for mean percentage of search strategy within the groups, Aβ animals showed increased preference for non-spatial search strategies. However, saline animals did not show differences from chance for both strategies. This apparently contradictory outcome might be due to a tendency to randomization of safe place search towards the end of the session, because the escape box is no longer present (Harrison et al., 2006). Indeed, in line with previous findings, saline animals changed preference across the probe session, and preferred spatial strategies as first choice as opposed to non-spatial strategies as last choice. Conversely, Aβ animals persevered on the non-spatial strategies, showing preference for this type of navigation towards the end of the probe session as well (see Table 1).

The Barnes maze has been used in studies of spatial learning and memory in transgenic models of AD (Pompl et al., 1999; Reiserer et al., 2007; O’Leary and Brown, 2009). Pompl et al. (1999) suggested that transgenic animals have difficulty to use spatial reference memory to reach a specific place (Chapman et al., 1999; Chen et al., 2000; Janus, 2004). This outcome resembles the memory deficit found in AD patients (Kaskie and Storandt, 1995) due to hippocampal function impairment (McNaughton et al., 1989; Poucet et al., 1991; McDonald and White, 1993). Rodriguez et al. (2013) demonstrated that APOE4 (an AD risk allele) mice showed delayed learning and impaired retrieval in the Barnes task. These animals also showed reduction in length and density of dendritic spines in the medial entorhinal cortex, and these alterations were related to early-impaired spatial cognition. Prut et al. (2007) showed that the AD transgenic mice APP23 had increased latency to reach the target and number of errors throughout the trainings in the Barnes maze, along with plaque deposition in the hippocampus and neocortex. Moreover, these animals had delays to switch from non-spatial to spatial strategies in the course of training. In the present study, we did not observe alterations during the training sessions of the Barnes maze, probably due to the mild nature of the cognitive deficit induced by our protocol. Indeed, we only observed spatial retrieval impairment in the probe trial.

In line with the deficit in the retrieval of spatial information, Janus (2004) found that AD transgenic mice (TgCRND8) did not use spatial strategies in the Morris water maze; they learned to reach the goal using a non-spatial strategy. Hamm et al. (2017) have also studied the transgenic TgCRND8 model, and showed an impaired association between object and place in a hippocampal dependent-task. In general, these previous studies suggest that the impairment in learning and retrieval of memory in AD models are due to deficits in the use of spatial strategies promoted by Aβ peptides increase and/or accumulation. In line with these findings, the results reported here show subtle alterations in spatial performance and the preferential use of non-spatial strategies after Aβ peptides infusion, suggesting a similarity with the early stages of AD.

Human versions of rodent spatial mazes in virtual environments have been used for detection of spatial memory deficits induced by hippocampal damage (Skelton et al., 2000; Moffat et al., 2001; Astur et al., 2002; Bohbot et al., 2004; Kumaran et al., 2007; Bartsch et al., 2010; Goodrich-Hunsaker et al., 2010). These investigations of spatial navigation in humans could allow early detection of dementia, which is relevant for early therapeutic intervention.

Nevertheless, similar to rodent tasks, the continuous exposition to such tasks diminishes the possibility of impairment detection (Jansen et al., 2010). On the other hand, the delayed retrieval assessment allows the detection of the episodic memory deficit (Albert, 1997; Foster, 1999). In this respect, contrary to the results described in the present study, Hamm et al. (2017) did not verify an impairment in the Barnes maze performance. This discrepancy is probably due to the reduced number of training trials and increased retrieval delay in our study. Thus, these protocol modifications allowed the detection of a spatial deficit similarly to the delayed retrieval protocol used in humans for the study of early impairments in AD.

The detailed etiology of Alzheimer remains unknown, although a great amount of evidence points to Aβ peptides as contributing factors to the neuronal dysfunctions and cognitive deficits of the disease (Braak and Braak, 1991; Dickey et al., 2003, 2004; Palop et al., 2003; Eustache et al., 2004; Sadek et al., 2004). The protocol of Aβ infusion used here induced a subtle cognitive deficit that was specific to spatial aspects, possibly compatible with prodromic characteristics of AD. Animals under Aβ treatment showed some extent of retrieval, but using non-spatial strategies. Further, these animals persevered on non-spatial choices until the end of the probe session. The results indicate that a task that can be solved by both spatial and non-spatial strategies is sensitive to detect deficits that could go unnoticed otherwise. The detection of subtle changes on cognition in an animal model can be useful to investigations of mechanisms as well as tests of potential neuroprotective approaches. We suggest that this approach is potentially useful to the study of the initial memory deficits in early AD.

## Author Contributions

PTM, RHL and RHS designed the research and wrote the article. PTM, ACQA, YSRM, LEMB and CLCC collected and analyzed the data. MRC and AMR contributed with theoretical and technical support.

## Conflict of Interest Statement

The authors declare that the research was conducted in the absence of any commercial or financial relationships that could be construed as a potential conflict of interest.
